# PRMT3 promotes tumorigenesis by methylating and stabilizing HIF1α in colorectal cancer

**DOI:** 10.1038/s41419-021-04352-w

**Published:** 2021-11-09

**Authors:** Xin Zhang, Kexin Wang, Xingbo Feng, Jian Wang, Yali Chu, Chunmeng Jia, Qingsi He, Cheng Chen

**Affiliations:** 1 Department of General Surgery, Qilu Hospital of Shandong University, 107 West Wenhua Road, Jinan, China; 2 Department of General Surgery, Central Hospital of Zaozhuang Coal Mining Group, Shandong Province, Zaozhuang, China; 3 Department of General Surgery, Jinan integrative medicine hospital, 8 east Wenyuan street, Jian, China

**Keywords:** Gastrointestinal cancer, Cell biology

## Abstract

Abnormal angiogenesis occurs during the growth of solid tumors resulting in increased vascular permeability to fluids and metastatic cancer cells. Anti-angiogenesis therapy for solid tumors is effective in the treatment of cancer patients. However, the efficacy of anti-angiogenesis therapy is limited by drug resistance. The findings of the current study showed that HIF1α R282 is methylated by PRMT3, which is necessary for its stabilization and oncogene function. Analysis showed that PRMT3-mediated tumorigenesis is HIF1α methylation-dependent. A novel therapeutic molecule (MPG-peptide) was used to inhibit HIF1α expression. These findings provided information on PRMT3 signaling pathway and HIF1/VEGFA signaling pathway and offer a novel therapeutic strategy for colorectal cancer, mainly for treatment of anti-angiogenesis resistance patients.

## Background

Angiogenesis is one of the hallmarks of cancer, and is characterized by sprouting of new blood vessels from pre-existing blood vessels [1, 2]. Tumor growth is characterized by insufficient supply of nutrients and oxygen, and poor clearance of metabolic waste [1, 3]. Hypoxia promotes tumor angiogenesis, resulting in malignant phenotype and aggressive tumor behavior [4, 5]. Pathological angiogenesis is driven by abnormal expression of pro-angiogenic factors, and solid tumor vessels are tortuous, disorganized, and permeable [4, 6]. Abnormal angiogenesis of solid tumors increases vascular permeability, enhances high interstitial fluid pressure, and reduces blood perfusion and oxygenation [2, 7, 8].

Hypoxia-induced factor 1α (HIF1α) is a pro-angiogenic factor characterized by abnormal overexpression under low oxygenation levels (hypoxia) in tissues, promoting expression of several pro-angiogenic factors [5, 9, 10]. Under normoxia, HIF1α is hydrolyzed by prolyl hydroxylase domain protein 2 (PHD2). Hydrolyzed-HIF1α can be poly-ubiquitinated by Von Hippel-Lindau (VHL) complex, and the poly-ubiquitinated-HIF1α is further degraded by proteasome [11–13]. However, under hypoxia, PHD2 loses its enzyme activity, and HIF1α escapes from degradation, binds to DNA with its co-activators and promotes transcription of genes, such as VEGFA, PDGF-B, erythropoietin (EPO), and GLUT-1 [14–17].

Protein arginine methyltransferases 3 (PRMT3) is a type-I PRMT family member, with a unique N-terminal C_2_H_2_ zinc finger motif and is localized in the cytoplasm [18, 19]. PRMT3 is related to ribosome small subunit and was first reported in fission yeast [20]. Although PRTM3 is widely distributed, only a few studies have explored its biological function in tumorigenesis.

HIF1α expression level is regulated by multiple posttranslational modifications, such as hydroxylation, acetylation, ubiquitination, phosphorylation, and lysine methylation [21–24]. The current study explored a novel HIF1α modification through arginine methylation. The findings indicate that PRMT3 is upregulated in colorectal cancer (CRC) tissues compared with adjacent normal tissue and its expression is negatively correlated with overall survival time of patients. Furthermore, analysis showed that R282 of HIF1α is asymmetrically di-methylated by PRMT3, and R282 asymmetric di-methylation of HIF1α decreases its HIF-1α poly-ubiquitination level, but not its hydroxylation level. Moreover, the findings showed that PRMT3-mediated tumorigenesis is HIF1α R282 methylation-dependent. Deletion of HIF1α R282 methylation significantly inhibits tumor progression and angiogenesis. These findings indicate that HIF1α is a novel target for colorectal tumor treatment.

## Material and methods

### Antibodies, cell lines, and reagents

RKO, LoVo, HEK293T, and HUVEC cell lines were purchased from the American Type Culture Collection (ATCC, Manassas, VA, USA). Antibodies against PRMT3 (Abcam; ab191562), GAPDH (Abcam; ab9485), H4R3me2a (Abcam; ab194683), histone 4 (Abclone; A1131), VEGFA (Abclone; A12303), HIF1α (cell signaling technology; #36169); HIF1β (cell signaling technology; #3718), flag-tag (cell signaling technology; #14793), stat3 (cell signaling technology; #9139), Y705-p-stat3 (cell signaling technology; #9145), β-actin (cell signaling technology; #3700) were used in the current study. SGC707 (HY-19715) was obtained from MedChemExpress company. All cell lines were authenticated by STR profiling and there was no mycoplasma contamination.

### Enzyme-Linked Immunosorbent Assay (ELISA)

ELISA kit (RK00023) was purchased from Abclone company. ELISA assays were performed according to the manufacturer’s instructions.

### Wound healing assay

Pre-treated cells were seeded in a 6-well plate and cultured to 95% confluency. A linear wound was made on the plate using a sterile pipette tip. The wound diameter was then determined at the indicated times.

### Transwell assay

Transwell chambers (8 mm) were purchased from Costar, Cambridge, United Kingdom. 4 × 10^4^ (for normoxia) or 10 × 10^4^ (for hypoxia) cells in 250 µL serum-free medium were seeded in the upper chamber, and 400 µL conditional mediums were added to the lower chamber. After culturing for 12–16 h, cells were fixed with 4% paraformaldehyde and stained with 0.5% crystal violet. Cell number was determined using a microscope.

### CCK8 assays

CCK8 kit (RM02823) was purchased from Abclone company. CCK8 assays were performed according to the manufacturer’s instructions.

### Western blots analysis and Immunoprecipitation (IP)

Pre-treated cells were obtained and washed twice with cold PBS. The cells were then lysed using NP-40 lysis buffer for 30 min at 4 °C. Protein concentration was determined using bicinchoninic acid assay kit (Thermo Fisher Scientific). Proteins were separated by electrophoresis using a premade sodium dodecyl sulfate-polyacrylamide minigel (Tris-HCL SDS-PAGE) and then transferred to PVDF membranes. The membranes were incubated with primary antibodies overnight at 4 °C and then further incubated with secondary antibodies. The signal was detected using the chemiluminescence method. For immunoprecipitation, extracted proteins were incubated with identified antibodies overnight at 4 °C and then centrifuged with protein A/G beads for 2–4 h at 4 °C. The beads were then washed thrice using NP-40 lysis buffer, and western blot assays were performed.

### Cell cycle analysis

Cells were washed with cold PBS, and then fixed in 80% ethanol overnight at −20 °C. Cells were stained with PI at room temperature for 5–10 min. Cell cycle distribution was measured using BD Biosciences System.

### Cell apoptosis analysis

Annexin V-FITC/PI staining kit were purchased from Sigma-Aldrich. Cell apoptosis assays were conducted according to the manufacturer’s instructions, followed by flow cytometry to examine apoptosis (BD Biosciences).

### Quantitative Real-time PCR

Total RNA was isolated using TRIzol (Invitrogen, Carlsbad, CA, USA) according to manufacturer’s protocol. The RNA was reverse transcribed using ABScript II kit (Abclone; RK20402) to obtain cDNA. Real-time PCR assays were conducted and RT-PCR kit (Abclone; RK21203) and analyzed using Multi-color Real-Time PCR Detection System (Bio-Rad, Hercules, CA, USA). Primers used are listed as followed: PRMT3 (forward: 5-GTACCCTTCTCATACCCCAATGG-3;backward:5-GACGAGCAGGTTCTGACATCT-3),HIF1α(5-GAACGTCGAAAAGAAAAGTCTCG-3;backward:5-CCTTATCAAGATGCGAACTCACA-3),VEGFA(5-AGGGCAGAATCATCACGAAGT-3;backward:5-AGGGTCTCGATTGGATGGCA-3),beta-actin(5-CATGTACGTTGCTATCCAGGC-3; backward: 5-CTCCTTAATGTCACGCACGAT-3).

### Colorectal tumor clinic samples

Primary tumor and adjacent normal tissues were harvested from colorectal cancer patients who underwent surgical operation without preoperative chemoradiotherapy at Qilu Hospital of Shandong University and samples were stored at −80 °C. The inclusion and exclusion criteria are listed as followed:1). primary tumor is colorectal cancer. 2). no preoperative radiotherapy and chemotherapy. 3). patients are firstly diagnosed with colorectal cancer, without other malignancy. 4). the tumor of patients can be cut off. The informed consents were obtained from all patients. This study was approved by Shandong University Ethical Review Committee.

### Construction of lentiviral-infected cell lines

pLKO-AS3w-encoding-identified gene, MD2-G, and PPAX three-pack system were used to generate a high-expression virus. In addition, PLKO.1, MD2-G, and PPAX three-pack system were used to silence gene expression in the virus. Sequences for shPRMT3 were as follows: shPRMT3#1: 5′-CCTTGTGGTATTAAGCATATA-3′; shPRMT3#2: 5′- CGTGACCCTCACGTTGAATAA -3′; shPRMT3#3: 5′-CCTTGGGAGAAAGAAGAGTA-3′. Sequences for SgRNAs for HIF1α knockout virus were as follows: 5′-CTCGAGATGCAGCCAGATCT-3′; 5′-CCATCAGCTATTTGCGTGTG-3′; 5′-TAACTCAGTTTGAACTAAC-3′. Cells were transfected with virus overexpressing or with silenced genes. Puromycin was used to select out infected cells after incubation for 48 h.

### Immunohistochemistry (IHC)

Immunohistochemistry assays were conducted as described in previous studies [25]. Two independent professors assessed IHC staining, and IHC staining score was calculated using IRS system. Percentage of positively stained tumor cells was scored as follows: 1 (<10%), 2 (10–50%), 3 (50–75%), and 4 (>75%). Staining intensity was scored from 0 to 3 with 0 indicating no staining; 1 indicating weak staining (light yellow); 2 representing moderate staining (yellow-brown); 3 representing strong staining (brown). Staining score was calculated by multiplying the score of the percentage of positive tumor cells and the staining intensity and it ranged from 0 to 12.

### Animal study

This study was approved by Shandong University Ethical Review Committee. Four-week-old female animals were purchased from Beijing Huafukang Bioscience Company. The mice were randomized into groups and there was five mice in every group. Pre-treated cells suspended in 100 µL of Matrigel were injected into the subcutaneous layer of nude mice. Tumor volume was determined every four days. Mice were sacrificed after one month and tumors collected and the tumor volume and weight determined.

### Statistical analysis

Statistical analysis was performed using SPSS 20.0 software. Before statistical analysis, the homogeneity of variance between groups was tested. Student’s *t* test was used to determine differences between two groups or ANOVA was used to determine differences among multiple groups. *P* < 0.05 was considered statistically significant.

## Result

### PRMT3 was upregulated and high-expression level was related with poor overall survival time in colorectal cancer

**Fig. 1:**
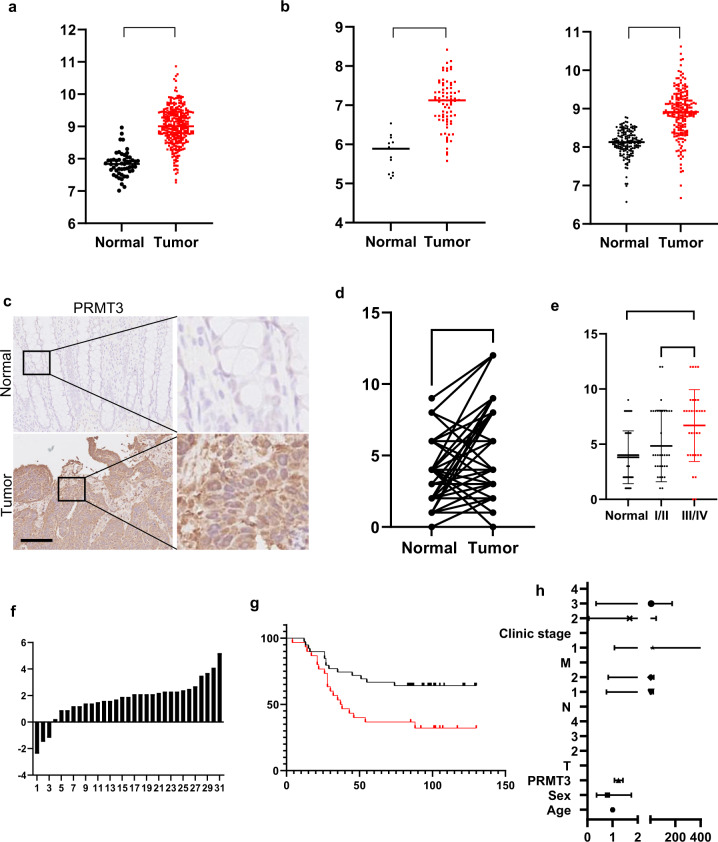
PRMT3 functioned as an oncogene in colorectal cancer. **a**, **b** Analysis of PRTM3 expression level in colorectal cancer tissue or normal colorectal tissue using TCGA (**a**) database, GSE24550 and GSE87211(**b**) datasets; **p < 0.05. **c** IHC analysis of PRMT3 expression level in 69 pairs of colorectal tumor tissues and their adjacent normal tissues, representative images are shown; scale = 100 µm. **d**, **e** Statistical analysis of PRMT3 expression level in 69 pairs of colorectal tumor tissues and their adjacent normal tissues, paired Student’s *t* test for image (**d**) and unpaired Student’s *t* test for image (**e**); ***p < 0.05. **f** Statistical analysis of PRMT3 expression level in 31 pairs of colorectal tumor tissues and their adjacent normal tissues using real-time PCR assays. **g** Kaplan–Meier plot of overall survival: patients were grouped based on the IHC score for PRMT3 expression; *p* = 0.0215. **h** Multivariate analysis to evaluate the association of PRMT3 expression with prognosis of CRC patients in the presence of other clinical variables. Student’s *t* test was employed for statistical analysis.

To explore the biological role of PRMT3 in tumorigenesis, data retrieved from The Cancer Genome Atlas (TCGA) and GEO databases were analyzed. The findings showed that PRMT3 expression level in colorectal tumor tissue was significantly higher compared with that of normal colorectal tissues (Fig. 1a, b). In addition, analysis using GEPIA database indicated that PRMT3 was upregulated in various cancers (Supplementary Fig. 1a). A total of 69 pairs of colorectal tumor tissues and their adjacent normal tissues were thus obtained. IHC and RT-PCR assays were carried out to explore the expression level of PRMT3 in colorectal cancer. Results showed that PRMT3 was significantly upregulated in colorectal tumor tissues compared with the expression level in adjacent normal tissues (Fig. 1c–f). Notably, patients with high PRMT3 expression levels had worse overall survival times compared with those with lower PRMT3 expression levels (Fig. 1g). Analysis of data retrieved from human protein atlas showed that liver and pancreatic cancer patients with high PRMT3 expression levels presented with worse overall survival times compared with patients with low expression level (Supplementary Fig. 1b). Furthermore, multivariate analysis indicated that PRMT3 expression level was an independent risk factor of overall survival time of patients (Fig. 1h). These findings implied that PRMT3 plays a role as a pro-tumorigenesis factor in tumorigenesis.

### PRMT3 induced cancer cell VEGFA expression

**Fig. 2:**
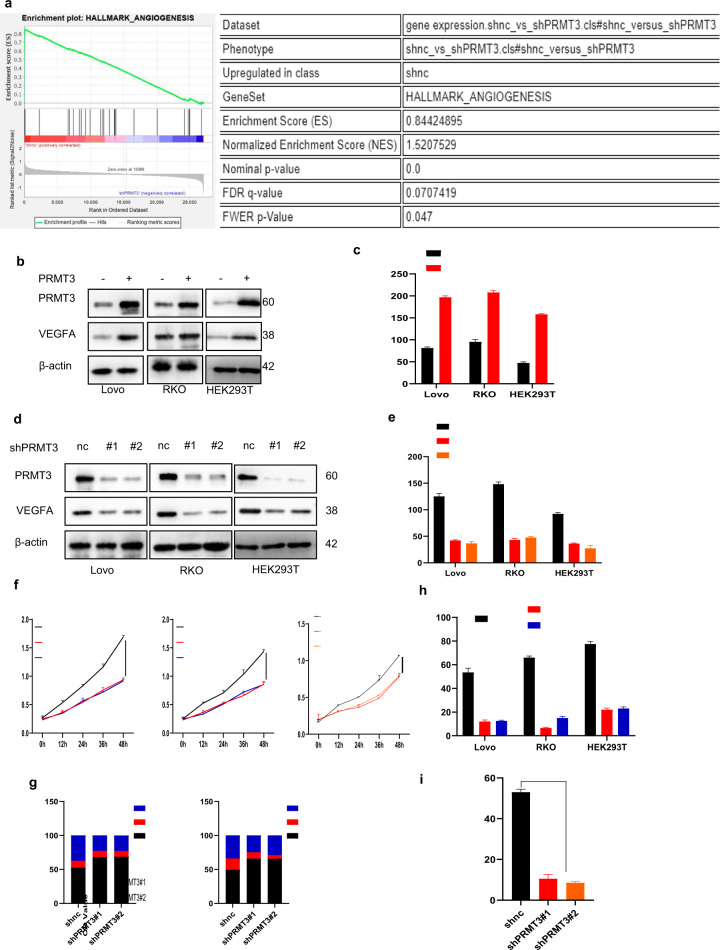
PRMT3 enhanced VEGFA expression. **a** Transfecting Lovo, RKO and HEK293T cells with vector or PRMT3 plasmid, immunoblots analysis for identified proteins expression level. **b** Transfection of Lovo, RKO and HEK293T cells with vector or PRMT3 plasmid, ELISA analysis of VEGFA expression level; **p < 0.05. **c** Silencing PRMT3 expression in Lovo, RKO and HEK293T cells using lentivirus, immunoblots analysis for identified proteins expression level. **d** Silencing PRMT3 expression level in Lovo, RKO and HEK293T cells using lentivirus, ELISA analysis of VEGFA expression; **p < 0.05. **e** Culturing HUVECs with conditional mediums, CCK8 analysis to investigate the proliferation of HUVECs. **p < 0.05. **f** Culturing HUVECs with conditional mediums, PI analysis of cell cycle. **g** Culturing HUVECs with conditional mediums, statistical analysis of transwell assays to investigate the migration of HUVECs (representative images were shown in Supplementary Fig. 2. j); **p < 0.05. **h** Culturing HUVECs with conditional mediums, statistical analysis of tube formation assays to investigate tuber formation ability of HUVECs (representative images were displayed in Supplementary Fig. 2. **m**); **p < 0.05. Student’s *t* or ANOVA test was utilized for statistical analysis. All immunoblots were conducted three times, affording same results.

To further explore the detailed mechanism in PRMT3-mediated tumorigenesis, RNA sequencing was performed using LoVo cells expressing shnc or shPRMT3. The findings showed that angiogenesis signaling pathway was significantly activated in LoVo cells ectopic expressing shnc compared with shPRMT3 cells. In addition, analysis of GSE87211 dataset showed similar results (Fig. 2a, Supplementary Fig. 2a, and Supplementary Table 1). Analysis of several datasets indicated that PRMT3 expression level was positively correlated with VEGFA expression level (Supplementary Fig. 2b, c). Results from western blot and ELISA assays showed that PRMT3 overexpression promotes VEGFA expression of cancer cells (Fig. 2b, c). Moreover, silencing PRMT3 inhibited VEGFA expression of cancer cells (Fig. 2d, e). Results from CCK8 assays showed that HUVEC cultured in conditional medium obtained from PRMT3 cells, had higher proliferation ability compared with HUVEC cultured in negative control conditional medium (Supplementary Fig. 2d). Similar results were observed for shPRMT3 cells (Fig. 2f). Besides, PI analysis showed that medium from cells ectopic expressing PRMT3 decreased the G0-G1 phase ratio but increased S phase ratio of HUVECs compared to medium from cells ectopic expressing Vector (Supplementary Fig. 2e), which was consistent with results from cells ectopic expressing shnc and shPRMT3 (Fig. 2g and Supplementary Fig. 3a). However, cell apoptosis analysis showed that there was no difference of apoptosis ratio of HUVECs cultured with different conditional mediums (Supplementary Fig. 3b). Further, the findings from transwell assays indicated that medium from PRMT3 cells significantly increased HUVEC migration ability compared with vector cells, and medium from loss-of-PRMT3 cells significantly inhibited HUVEC migration ability compared with negative control cells (Fig. 2h and Supplementary Fig. 3c–e). Results from tube formation assays showed that medium from gain-of-PRMT3 cells significantly promoted tube formation ability of HUVEC compared with medium from negative control cells (Supplementary Fig. 3f, g). Notably, consistent results were observed with loss-of-PRMT3 cells (Fig. 2i and Supplementary Fig. 4a). Furthermore, SGC707 was used to pharmacologically inhibit PRMT3 activity of cancer cells, and similar results were obtained (Supplementary Fig. 4b–d) [26]. These findings imply that PRMT3 activates angiogenesis signaling pathway and induces VEGFA expression in cancer cells.

### PRMT3 activated HIF1/VEGFA signaling pathway

**Fig. 3:**
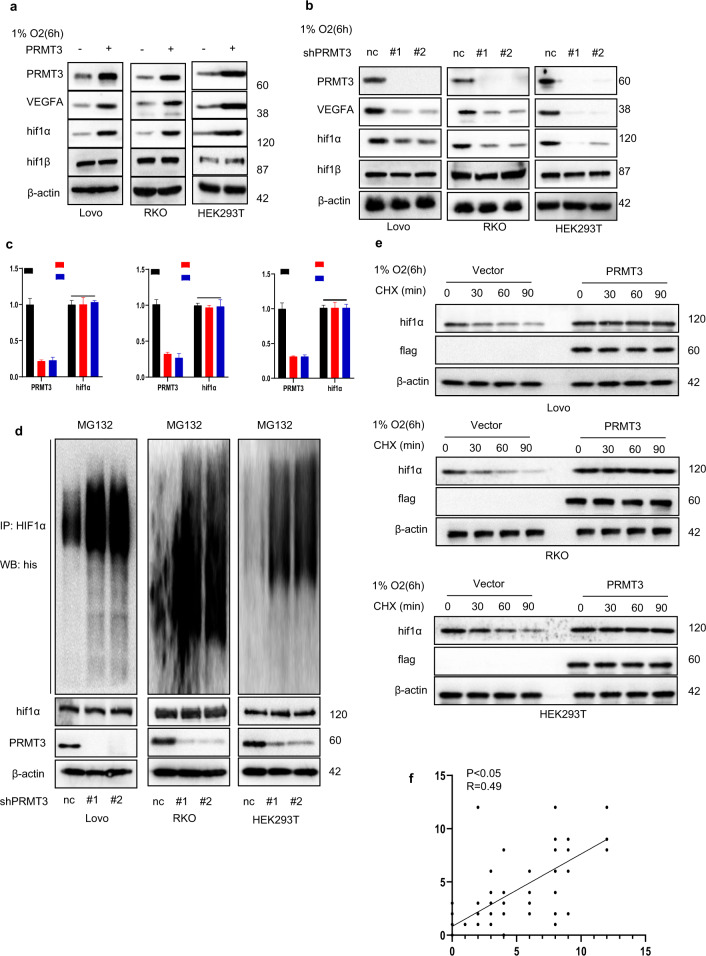
PRMT3 activated HIF1/VEGFA signaling pathway. **a** Transfecting Lovo, RKO and HEK293T cells with vector or PRMT3 plasmid, immunoblots analysis to investigate identified proteins expression level in hypoxia. **b** Silencing PRMT3 expression in Lovo, RKO and HEK293T, immunoblots analysis to investigate identified proteins expression level in hypoxia. **c** silencing PRMT3 expression level of Lovo, RKO and HEK293T cells using lentivirus, mRNAs expression level of the indicated proteins. **d** Silencing PRMT3 expression level in Lovo, RKO and HEK293T cells using lentivirus, Transfection of his-ubiquitination plasmid into cells, MG132 to inhibit endogenous HIF1α degradation; Isolating HIF1α from identified cells and then immunoblots analysis to assess HIF1α poly-ubiquitin level. **e** Cycloheximide (CHX) to inhibit protein synthesis, transfecting Lovo, RKO and HEK293T cell with vector or PRMT3 plasmid, immunoblots analysis to investigate the half-life of endogenous HIF-1α. **f** Statistical analysis of PRMT3 or HIF1α expression in colorectal cancer samples. Student’s *t* or ANOVA test was deployed for statistical analysis. All immunoblots were conducted three times, yielding identical results.

Further, the detailed mechanism of PRMT3-mediated VEGFA expression was explored. Unlike other members of PRMT family, PRMT3 is mainly localized in the cytoplasm, implying that PRMT3-mediated VEGFA expression might be independent of PRMT3-mediated histone modification [27]. Previous studies report that HIF1 complex and IL-6/STAT3 signaling pathway plays a crucial role in tumor angiogenesis, and STAT3 Y705 phosphorylation modulates IL-6/STAT3 signal pathway activation [11, 32-34]. However, The findings from western blot showed that PRMT3 overexpression significantly promoted HIF1α expression in hypoxia and normoxia but had no significant effect on the expression of STAT3 or phosphorylation of STAT3 Y705 (Fig. 3a and Supplementary Figs. 5a, 6a). Moreover, we used the CRISPR-Cas9 system to knock out PRMT3 gene in lovo cells. We found that Loss of PRMT3 or pharmacological inhibition of PRMT3 activity significantly inhibited HIF1α expression under hypoxia or nomoxia conditions and had no significant effect on the expression of STAT3 or phosphorylation of STAT3 Y705 (Fig. 3b and Supplementary Figs. 5b–d, 6b). RT-PCT results indicated that PRMT3 overexpression or silencing of PRMT3 had no significant effect on mRNA expression of HIF1α under hypoxia (Fig. 3c and Supplementary Fig. 5e). These findings implied that PRMT3 regulates HIF1α expression through post-transcriptional modifications. Poly-ubiquitin modification of HIF1α modulates HIF1α stabilization and expression level [11]. Therefore, the role of PRMT3 in modulation of HIF1α poly-ubiquitination was explored. Notably, upregulation of PRMT3 significantly inhibited HIF1α poly-ubiquitination, and downregulation of PRMT3 significantly increased HIF1α poly-ubiquitination (Fig. 3d and Supplementary Fig. 5f). In addition, treatment of cells with protein synthesis inhibitor (cycloheximide) showed that PRMT3 overexpression significantly increased the half-life of endogenous HIF-1a (Fig. 3e).

HIF1α is hydroxylated by PHDs. In normoxia condition, the enzyme activity of PHDs is significantly upregulated and cells exhibit increased HIF1α hydroxylation and decreased HIF-1 stabilization [11]. Notably, PRMT3 overexpression did not decrease HIF-1α hydroxylation level in normoxia condition (Supplementary Fig. 5g). Similar results were obtained in cells expressing ectopic shnc or shPRMT3 (Supplementary Fig. 5h). Moreover, PRMT3 expression level of colorectal tumor tissues was positively correlated with HIF1α expression level of colorectal tumor tissues (Fig. 3f and Supplementary Fig. 5i). These findings indicated that PRMT3 modulates HIF1α/VEGFA signaling pathway.

### PRMT3-mediated VEGFA expression depends on HIF1α

**Fig. 4:**
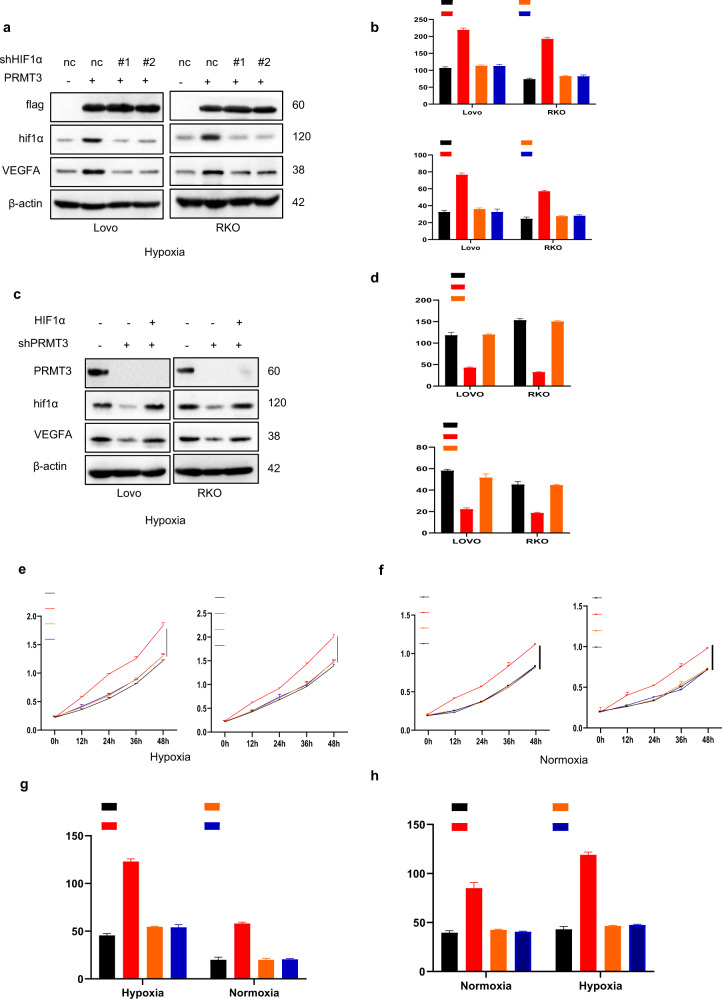
PRMT3 regulated VEGFA expression dependent on HIF1α. **a** Immunoblots analysis to investigate the identified protein expression level of cells ectopic expressing vector+shnc, PRMT3 + shnc, PRMT3 + shHIF1α#1, and PRMT3 + shHIF1α#2 in hypoxia. **b**. ELISA analysis to investigate VEGFA level of conditional mediums from cells ectopic expressing vector+shnc, PRMT3 + shnc, PRMT3 + shHIF1α#1, and PRMT3 + shHIF1α#2 in normoxia and hypoxia. **c** Immunoblots analysis investigates the identified protein expression level of cells ectopic expressing vector+shnc, shPRMT3+shnc, and shPRMT3 + HIF1α in hypoxia. **d** ELISA analysis investigates the VEGFA level of conditional mediums from cells ectopic expressing vector+shnc, shPRMT3+shnc, and shPRMT3 + HIF1α in normoxia hypoxia. **e**–**f** CCK8 assays to investigate the proliferation ability of HUVECs treated by conditional medium from cells ectopic expressing vector+shnc, PRMT3 + shnc, PRMT3 + shHIF1α#1, and PRMT3 + shHIF1α#2 in hypoxia (**e**) and normoxia (**f**). **g–h** HUVECs treated with conditional mediums from cells ectopic expressing vector+shnc, PRMT3 + shnc, PRMT3 + shHIF1α#1, and PRMT3 + shHIF1α#2 to investigate HUVECs migration (**g**), and tube formation ability (**h**) in normoxia and hypoxia; Statistical analysis of transwell assays (**g**), and tube formation assays (**h**). Student’s *t* or ANOVA test was used for statistical analysis. All immunoblots were conducted three times, producing identical results.

Four different kinds of LoVo and RKO cell lines expressing ectopic vector + shnc, PRMT3 + shnc, PRMT3 + shHIF1α#1, and PRMT3 + shHIF1α#2 were constructed. The finding from western blot and ELISA assays showed that increased expression of VEGFA induced by PRMT3 was reversed by silencing HIF1α expression in hypoxia and normoxia condition (Fig. 4a, b and Supplementary Fig. 6a). Three types of LoVo and RKO cell lines expressing ectopic vector + shnc, shPRMT3+vector, and shPRMT3 + HIF1α were constructed. The findings showed that a decrease in the expression level of VEGFA caused by silencing of PRMT3 was reversed by upregulation of HIF1α in hypoxia and normoxia condition (Fig. 4c, d and Supplementary Fig. 6b). Moreover, The results from CCK8 analysis, transwell assays, and tube formation assays showed that medium from cells expressing ectopic PRMT3 presenting high HUVECs pro-proliferation, pro-migration, and pro-tube formation ability was reversed by silencing HIF1α expression (Fig. 4e–h and Supplementary Fig. 6c, d). Consistently, HIF1α overexpression was abrogated by medium from cells expressing ectopic shPRMT3 with low HUVECs proliferation, migration, and tube formation ability (Supplementary Fig. 7a–e). These findings indicated that PRMT3 induced VEGFA expression by stabilizing HIF1α and activating HIF1/VEGFA signaling pathway and PRMT3-mediated VEGFA overexpression depends on HIF1α expression.

### PRMT3 methylated HIF1α at arginine 282 site

**Fig. 5:**
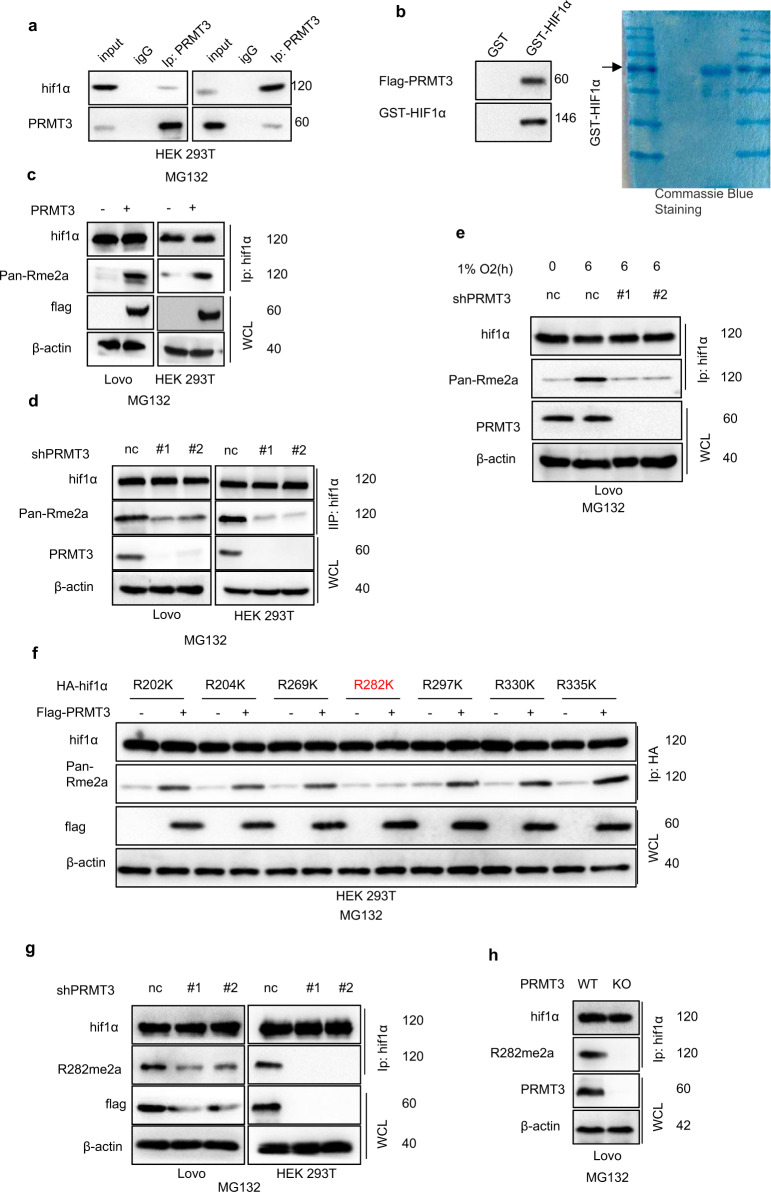
HIF1α R282 was methylated by PRMT3. **a** whole-cell lysis was collected for Co-IP analysis using identified antibodies, followed by immunoblots analysis. **b** Incubating GST-HIF1α and PRMT3 protein, followed by GST-pulldown and immunoblots analyses. **c** Co-IP and immunoblots analysis to investigate the asymmetric demethylation level of HIF1α of cells ectopic expressing vector or PRMT3. **d** Silencing PRMT3 expression using lentivirus, Co-IP and immunoblots analysis to investigate the asymmetric demethylation level of HIF1α. **e** Silencing PRMT3 expression using lentivirus; Co-IP and immunoblots analyses to investigate HIF1αasymmetric demethylation level with or without hypoxia. **f** HEK 293T cells were respectively transfected with 7 HA-tag methylation-deficient variants of HIF1α and vector or PRMT3 plasmid; Co-IP and immunoblots analyses to investigate HIF1α asymmetric demethylation level under MG132 treatment. **g** Silencing PRMT3 expression using lentivirus; Co-IP and immunoblots analysis to assess R292me2a level of HIF1α. **h** Knocked out PRMT3 expression using lentivirus; Co-IP and immunoblots analysis to assess R292me2a level of HIF1α. All immunoblots were conducted three times, affording same results.

Analysis showed a physical interaction between HIF1α and PRMT3 in HEK 293 T and LoVo cells under normoxia conditions (Fig. 5a and Supplementary Fig. 8a). GST-pulldown analysis showed a direct binding between HIF1α and PRMT3 (Fig. 5b). Notably, hypoxia enhanced interaction between HIF1α and PRMT3 (Supplementary Fig. 8b). Moreover, methyltransferase-deficiency PRMT3 E335Q overexpression showed no significant effect on HIF1α expression (Supplementary Fig. 8c). Further analysis was conducted to explore whether PRMT3 methylates HIF1α arginine residues. Pan-asymmetric dimethylarginine antibody (pan-Rme2a) or pan- mono-methyl arginine antibody (pan-Rme) was used to detect asymmetric dimethyl /mono-methyl arginine level of HIF1α in cells. The findings showed that PRMT3 overexpression significantly increased asymmetric dimethylarginine level of HIF1α but not mono-methyl arginine level of HIF1α (Fig. 5c and Supplementary Fig. 8d). Notably, hypoxia increased HIF1α asymmetric dimethylarginine level but not mono-methyl arginine level (Supplementary Fig. 8e). Methyltransferase-deficiency PRMT3 E335Q had no significant effect on asymmetric dimethylarginine level of HIF1α (Supplementary Fig. 8f). Furthermore, silencing of PRMT3 or pharmacological inhibition of PRMT3 activity significantly reduced the asymmetric dimethylarginine level of HIF1α (Fig. 5d and Supplementary Fig. 8g). Moreover, loss of PRMT3 abrogated the upregulated asymmetric dimethylarginine level of hypoxia-mediated HIF1α (Fig. 5e). These findings implied that PRMT3 regulates HIF1α asymmetric dimethylarginine level, and these effects depend on enzymatic activity of PRMT3.

Further analysis was conducted to explore which arginine residues of HIF1α were methylated by PRMT3. Analysis was conducted to determine where PRMT3 binds on HIF1α protein. Four plasmids separately containing 1–200aa, 201–400aa, 401–600aa, and 601-terminal of HIF1α CDS sequences were constructed. The four HA-tag separate plasmids were transfected separately into HEK293T cells. Co-IP analysis showed that 201–400 aa of HIF1α CDS sequences were bound to PRMT3 (Supplementary Fig. 8h). In addition, deletion of 201–400 aa of HIF1α CDS sequences abrogated interaction between HIF1α and PRMT3 (Supplementary Fig. 8i). These findings imply that PRTM3 methylates HIF1α at its 201–400aa CDS sequence. Further analysis was performed to explore which arginine residues were methylated by PRMT3. The HIF1α 201–400 CDS sequence comprised seven arginine residues. Seven methylation-deficient variants of HIF1α (R202K, R204K, R269K, R282K, R297K, R330K, and R335K) were constructed. Seven HA-tag methylation-deficient variants of HIF1α and vector or Flag-tag PRMT3 were co-transfected into HEK293T cells. The findings showed that HIF1α R282K methylation-deficient variant abrogated PRMT3-mediated HIF1α asymmetric dimethylarginine methylation upregulation (Fig. 5f). To further validate PRMT3-mediated arginine methylation site of HIF1α, an R282 specific asymmetric dimethylarginine antibody (R282me2a) was generated (Supplementary Fig. 8j). Analysis using R282me2a antibody showed that HIF1α R282 asymmetric di-methylation was upregulated by ectopic expression of PRMT3, but not methyltransferase-deficiency PRMT3 E335Q (Supplementary Fig. 8k–m). Consistently, downregulation of PRMT3 or pharmacological inhibition of PRMT3 activity significantly inhibited HIF1α R282 asymmetric di-methylation (Fig. 5g, h and Supplementary Fig. 8n). In summary, these results indicated that PRMT3 asymmetrically di-methylates HIF1α at arginine 282.

### RMT3 regulated HIF1/VEGA signaling pathway, and tumor angiogenesis is modulated by HIF1α arginine methylation

**Fig. 6:**
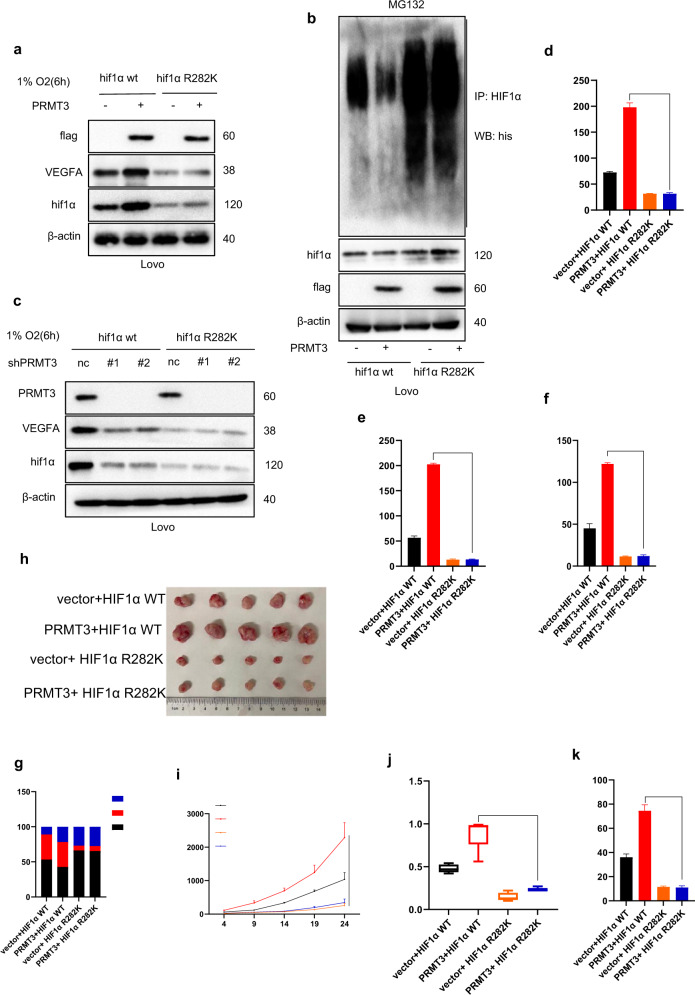
HIF1α R282 methylation was essential for PRMT3-mediated tumorigenesis. **a** Transfecting vector or PRMT3 into Lovo cells stably expressing HIF1α WT or HIF1α R282K mutant; Immunoblots analysis to investigate identified proteins expression level under hypoxia. **b** Transfecting vector or PRMT3 into Lovo cells stably expressing HIF1α WT or HIF1α R282K mutant, all cells transfected with his-ubiquitination plasmid; Co-IP and immunoblots analyses to detect HIF1α poly-ubiquitin level. **c** Silencing PRMT3 expression level of Lovo cells stably expressing HIF1α WT or HIF1α R282K mutant using lentivirus; Immunoblots analysis to investigate identified proteins expression level under hypoxia. **d** Transfecting vector or PRMT3 into Lovo cells stably expressing HIF1α WT or HIF1α R282K mutant; ELISA analysis to assess the VEGFA level of conditional medium. **e**, **f** Collecting conditional mediums from identified cells, culturing HUVECs with conditional mediums; statistical analysis of transwell (**e**), and tube formation (**f**) assays to assay the migration, and tube formation ability of HUVECs in hypoxia. **g** Culturing HUVECs with conditional mediums; PI analysis of cell cycle. **h** Tumor images shown. **i** Tumor growth curve; **p < 0.05. **j** Scatter plot showing tumor weight; **p < 0.05. **k** Scatter plot showing microvessel density; **p < 0.05. Student’s *t* test was used for statistical analysis. All immunoblots were conducted three times, providing same results.

To explore PRMT3-mediated HIF1α methylation, endogenous HIF1α expression was knocked out in LoVo cells using lentivirus (Supplementary Fig. 9a). Two LoVo cell lines stably expressing ectopic HIF1α WT or HIF1α R282K mutant were then constructed. Western blot results showed an equal protein expression level of HIF1α in the two cell lines (Supplementary Fig. 9b). PRMT3 plasmid was transfected into LoVo cells. The results indicated that PRMT3 upregulated HIF1α and VEGFA expression, and decreased HIF1α poly-ubiquitin level in cells expressing HIF1α WT, but not in cells expressing HIF1α R282K mutant. In addition, expression level of HIF1α and VEGFA in cells expressing HIF1α R282K mutant was significantly lower compared with the levels in cells expressing HIF1α WT. HIF1α R282K mutant significantly promoted poly-ubiquitin level of HIF1α compared with that of HIF1α WT (Fig. 6a, b). Consistently, knockdown of PRMT3 or pharmacological inhibition of PRMT3 activity significantly decreased HIF1α and VEGFA expression level in cells expressing HIF1α WT, but not in cells expressing HIF1α R282K mutant (Fig. 6c). ELISA analysis showed that PRMT3 overexpression promoted VEGFA expression in cells expressing ectopic HIF1α WT, but not in cells expressing HIF1α R282K (Fig. 6d and Supplementary Fig. 9c). Furthermore, HUVEC was cultured using conditional media from vector+HIF1α WT, PRMT3 + HIF1α WT, vector+ HIF1α R282K, and PRMT3 + HIF1α R282K cells. CCK8, transwell, and tube formation analysis showed that conditional mediums from HIF1α WT cells, but not HIF1α R282K cells ectopic expressing PRMT3 promoted proliferation, migration, and tube formation of HUVEC compared with that from HIF1α WT cells ectopic expressing vector(Fig. 6e, f and Supplementary Fig. 9d–f). Moreover, PI analysis showed that conditional mediums from HIF1α WT cells ectopic expressing PRMT3 decreased the G0-G1 phase ratio but increased S phase ratio of HUVECs (Fig. 6g and Supplementary Fig. 9g). The four cell lines were subcutaneously administered into the back of 4-week-old male nude mice. All mice were sacrificed after 24 days. The results revealed that HIF1α WT cells grew faster and exhibited higher volume compared with HIF1α R282K. In addition, PRMT3 overexpression significantly promoted tumor growth in cells expressing HIF1α WT, but not in cells expressing HIF1α R282K (Fig. 6h–j). Moreover, The findings from IHC assays indicated that upregulation of PRMT3 significantly increased tumor angiogenesis in tumors induced by cells expressing HIF1α WT, but not cells expressing HIF1α R282K (Fig. 6k and Supplementary Fig. 9h). These findings indicated that PRMT3-mediated HIF1α R282 methylation played an important role in PRMT3-mediated tumor angiogenesis.

### Targeting HIF1α R282 methylation was a potential therapeutic strategy for HIF1α-driven colorectal cancer treatment

**Fig. 7:**
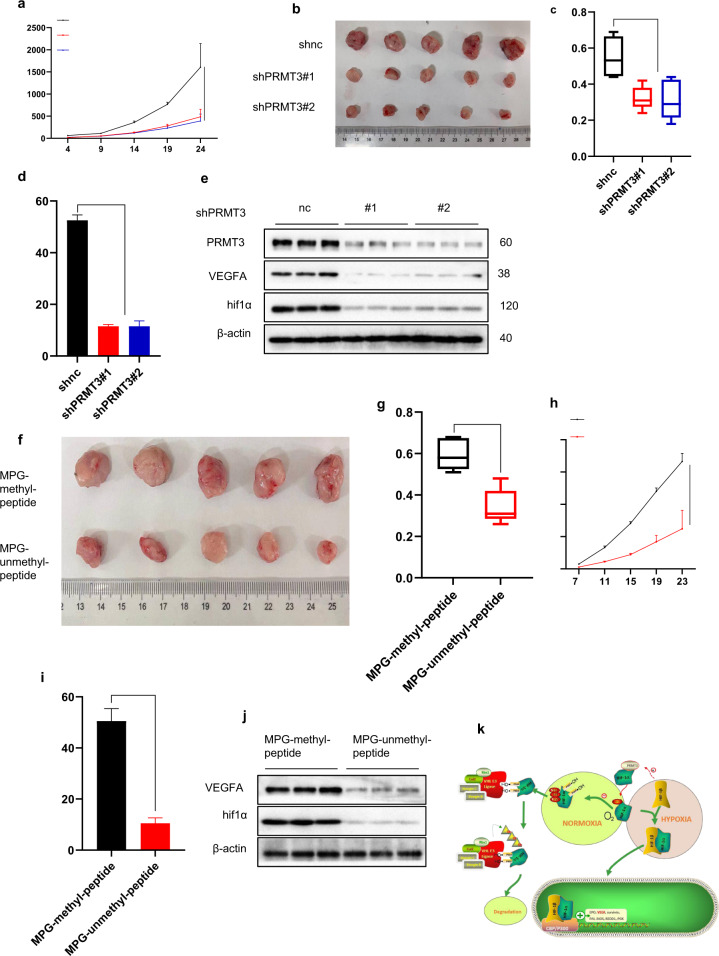
Loss of HIF1α R282 methylation inhibited tumor progress. **a**, **h** Tumor growth curve is shown; **p < 0.05. **b**, **f** Tumor images are shown. **c**, **g** Scatter plot showing tumor weight; **p < 0.05. **d**, **i** Scatter plot showing microvessel density; **p < 0.05. **e**, **j** Isolating proteins from tumor; immunoblots analysis to detect identified proteins expression level. **k** Summary of the mechanisms of HIF1α stabilization by PRMT3 via R282 methylation. Student’s *t* or ANOVA test was used for statistical analysis. All immunoblots were conducted three times with consistent results.

Based on upon findings, targeting HIF1α R282 methylation was a potential and effective treatment strategy. Therefore, three LoVo cell lines (shnc, shPRMT3#1, and shPRMT3#2) were subcutaneously injected into the back of 4-week-old male nude mice. Analysis showed that silencing of PRMT3 significantly inhibited tumor growth and tumor angiogenesis (Fig. 7a–d and Supplementary Fig. 10a). Moreover, western blot analysis showed that loss of PRMT3 significantly inhibited the expression of HIF1α and VEGFA (Fig. 7e).

Peptide drugs are potential therapeutic agents for cancer treatment [28]. A corresponding peptide with arginine asymmetrically demethylated was generated. A cell-penetrating peptide (MPG) was placed in the N terminal of the two peptides (Supplementary Fig. 10b) [28]. To explore whether MPG-non-methylation peptide inhibits HIF1α R282 methylation, cells were treated with MPG-non-methylation peptide or MPG-methylation peptide. The MPG-non-methylation peptide, depressed HIF1α R282 methylation and inhibited HIF1α and VEGFA expression in hypoxia whereas MPG-methylation peptide showed no significant effects (Supplementary Fig. 10c–e). HUVECs were cultured with medium from cells treated with MPG-non-methylation peptide or MPG-methylation peptide. CCK8 analysis showed that HUVECs cultured with medium from cells treated with MPG-non-methylation peptide had lower proliferation ability compared with HUVECs cultured with medium from cells treated with MPG-methylation peptide (Supplementary Fig. 10f). LoVo cells were subcutaneously injected into the back of 4-week-old male nude mice. Two days after injection, mice were treated with MPG loaded with methylated-peptide or non-methylated-peptide every three days. The results indicated that MPG-non-methylation peptide significantly inhibited tumor growth and tumor angiogenesis (Fig. 7f–i and Supplementary Fig. 10g). Moreover, western blot analysis showed that MPG-non-methylation peptide significantly inhibited the expression of HIF1α and VEGFA (Fig. 7j). In summary, these findings indicated that PRMT3 inhibition is a novel and potential therapeutic strategy for treatment of cancer patients.

## Discussion

Tumor-associated abnormal vascular vessel development increases vascular permeability to fluids and metastatic cancer cells [29]. Pharmacological inhibition of tumor angiogenesis promotes tumor starvation, induces cell death, restrains tumor progress, improves therapeutic effect, and increases overall survival time of patients [10]. HIF1α is induced by hypoxia and its expression is significantly upregulated after anti-angiogenic therapy, resulting in drug resistance [10].

A previous clinical study reported that HIF1α was significantly upregulated in bevacizumab resistant metastatic colorectal cancer, thus it is a potential biomarker of anti-angiogenesis resistance [25]. Posttranslational regulation of HIF1α is an important step for HIF1α expression and may provide a therapeutic target for modulating anti-angiogenesis resistance. Previous studies report that HIF1α expression is regulated by its hydroxylation, acetylation, ubiquitination, phosphorylation, and lysine methylation. The findings of the current study showed a novel modification of HIF1α, arginine methylation. The finding indicated that HIF1α R282 methylation promotes HIF1α stability. Notably, HIF1 R282 methylation inhibited its poly-ubiquitination, but not its hydroxylation level. Analysis using xenograft tumor assays showed that deletion of R282 methylation inhibited tumor progression.

PRMT3 is a member of PRMT family mainly localized in the cytoplasm under physiological conditions [27]. Previous studies report that PRMT3 enhances chemoresistance by upregulating ABCG2 expression and by inducing metabolic reprogramming in pancreatic cancer. The findings of the current study showed that PRMT3 was upregulated in CRC patients, was associated with poor overall survival of patients, and was an independent risk factor for overall survival of patients (Fig. 1). Analysis showed that PRMT3 regulates HIF1/VEGFA signaling pathway and tumor angiogenesis by methylating HIF1α R282 and promoting stability of HIF1α. In addition, xenograft tumor assays showed that PRMT3-mediated tumorigenesis was dependent on HIF1α R282 methylation, and cross-talks between PRMT3-mediated tumorigenesis and HIF1 signaling pathways are implicated in tumor angiogenesis.

Several studies have been conducted on pharmaceutical amino acid polypeptides for tumor treatment [28]. In the current study, MPG-unmethylated-peptide significantly inhibited HIF1/VEGFA signaling pathway activity in vitro. Xenograft tumor assays further showed that MPG-unmethylated-peptide therapeutically inhibited tumor progression (Fig. 7f), thus indicating a novel therapeutic strategy for colorectal tumor treatment.

## Conclusion

In summary, the findings of the current study show that PRMT3 was high expressed in colorectal cancer and the expression level was correlated with overall survival of patients. Further analysis showed that PRTM3 modulates HIF1/VEGFA signaling pathway by stabilizing HIF1α. In addition, PRMT3-mediated HIF1α R282 methylation played an important role in HIF1α stabilization. Analysis using xenograft tumor assays showed that HIF1α R282 methylation was important for its oncogene function (Fig. 7k). Moreover, analysis of the relationship between PRMT3 and HIF1/VEGFA signaling pathways showed that PRMT3 pro-tumorigenesis was HIF1α R282 methylation-dependent. These findings provide a novel pharmacological strategy (MPG-peptide) for inhibiting HIF1α expression, and which may be an effective therapeutic method for colorectal cancer treatment, mainly for anti-angiogenesis resistance patients.

## Supplementary information

supplementary figure legend

supplementary figure 1

supplementary figure 2

supplementary figure 3

supplementary figure 4

supplementary figure 5

supplementary figure 6

supplementary figure 7

supplementary figure 8

supplementary figure 9

supplementary figure 10

supplementary table 1

## Data Availability

All data and materials used in the study are available in the manuscript.
