# Supplementary material for: PRMT3 promotes tumorigenesis by methylating and stabilizing HIF1α in colorectal cancer
Source: Cell Death Dis. 2021 Nov 9;12(11):1066. doi: 10.1038/s41419-021-04352-w (PMC8578369; doi:10.1038/s41419-021-04352-w)
Supplement: Supplementary file 1 — supplementary figure legend [file 41419_2021_4352_MOESM1_ESM.docx]

Supplementary figure Legends

Figure S1. PRMT3 acted at an oncogene gene. **a**. PRMT3 expression level in different cancers using GEPIA database. **b**. Kaplan–Meier plot showing overall survival of liver cancer (upper) or pancreatic cancer (lower): patients were grouped based on PRMT3 expression level; **p<0.05.

Figure S2. PRMT3 promoted VEGFA expression in cancer cells. **a**. GSEA analysis using GSE87211 dataset. **b**. Analysis of the relationship between VEGFA and PRMT3 using GSE63216 and GSE24550 datasets; **p<0.05. **c**. Analysis of the relationship between VEGFA and PRMT3 in the TCGA dataset; **p<0.05. **d**. Culture of HUVECs with conditional mediums, CCK8 assay to investigate HUVECs proliferation ability; **p<0.05. **e**. Culturing HUVECs with conditional mediums, PI analysis of cell cycle. Student-t or ANOVA test was used for statistical analysis. All immunoblots were conducted three times, producing identical results.

Figure S3. PRMT3 promoted VEGFA expression in cancer cells. **a**. Culturing HUVECs with conditional mediums, PI analysis of cell cycle. **b**.Culturing HUVECs with conditional mediums, apoptosis analysis of cell cycle. **c-g**. Culture of HUVECs with conditional media, showing representative images of transwell assays or tube formation assays; **p<0.05. Student-t or ANOVA test was used for statistical analysis. All immunoblots were conducted three times, producing identical results.

Figure S4. PRMT3 promoted VEGFA expression in cancer cells. **a**. Culture of HUVECs with conditional media, showing representative images of tube formation assays. **b**. Cells treated with SGC707 (an inhibitor of PRTM3), immunoblots analysis to investigate identified protein expression level. **c**. Cells treated by SGC707; ELISA analysis to assess the VEGFA expression level of conditional mediums; **p<0.05. **d**. Cells treated by SGC707, culture of HUVECs with conditional mediums, CCK8 assay to investigate HUVECs proliferation ability; **p<0.05. Student-t or ANOVA test was used for statistical analysis. All immunoblots were conducted three times, producing identical results.

Figure S5. PRMT3 regulated HIF1/VEGFA signaling pathway. **a, g**. Cells transfected with vector or PRMT3 plasmid, immunoblots analysis to assess identified protein expression level. **b, h**. Silencing PRMT3 expression using lentivirus, immunoblots analysis to assess identified protein expression level. **c**. Cells treated by SGC707, immunoblots analysis to assess identified protein expression level. **d**. Knocking out PRMT3 expression using lentivirus, immunoblots analysis to assess identified protein expression level. **e**. Transfecting Lovo, RKO and HEK293T cells with vector or PRMT3 plasmid, mRNAs expression level of the indicated proteins. **f**. Transfecting Lovo, RKO and HEK293T cells with vector or PRMT3 plasmid, Transfection of his-ubiquitination plasmid into cells, MG132 to inhibit endogenous HIF1α degradation; Isolating HIF1α from identified cells and then immunoblots analysis to assess HIF1α poly-ubiquitin level. **i**. Showing representative images of IHC for colorectal cancer sample. Student-t or ANOVA test was used for statistical analysis. All immunoblots were conducted three times, providing same results.

Figure S6. PRMT3 regulated HIF1/VEGFA signaling pathwayPRMT3 regulated HIF1/VEGFA signaling pathway. **a**. Immunoblots analysis to investigate the identified protein expression level of cells ectopic expressing vector+shnc, PRMT3+shnc, PRMT3+shHIF1α#1, and PRMT3+shHIF1α#2 in normoxia. **b**. Immunoblots analysis investigates the identified protein expression level of cells ectopic expressing vector+shnc, shPRMT3+shnc, and shPRMT3+HIF1α in normoxia. c. Lovo cells transfected with vector or PRMT3, followed by Co-IP or immunoblots analysis. **c**. Culture of HUVECs with conditional mediums, showing representative images of transwell assays in normoxia and hypoxia. **d**. Culture of HUVECs with conditional mediums, showing representative images of tube formation assays in normoxia and hypoxia.

Figure S7. PRMT3 regulated HIF1/VEGFA signaling pathwayPRMT3 regulated HIF1/VEGFA signaling pathway. **a**. Culture of HUVECs with conditional mediums, showing representative images of transwell assays in normoxia and hypoxia; **p<0.05. **b, c**. Culture of HUVECs with conditional mediums, CCK8 assay to investigate HUVECs proliferation ability in normoxia(**f**) and hypoxia(**g**); **p<0.05. **d, e**. Culture of HUVECs with conditional mediums, showing representative images of tube formation assays in normoxia and hypoxia; **p<0.05. Student-t or ANOVA test was used for statistical analysis. All immunoblots were conducted three times, providing same results.

Figure S8. PRMT3 methylated HIF1α at R282. **a**. whole-cell lysis was collected for Co-IP analysis using identified antibodies, followed by immunoblots analysis. **b**. Lovo treated by hypoxia, MG132 to inhibit HIF1α degradation, whole-cell lysis was collected for Co-IP analysis using identified antibodies, followed by immunoblots analysis. **c**. HEK 293T cells transfected with vector, PRMT3 or PRMT3 E335Q, then treated by hypoxia, followed by immunoblots analysis. **d**. Lovo cells transfected with vector or PRMT3, followed by Co-IP or immunoblots analysis. **e, g**. Lovo treated with hypoxia (**e**) or SGC707(**g**), followed by Co-IP or immunoblots analysis. **f**. Co-IP and immunoblots analyses investigate the asymmetric demethylation level of HIF1α of cells ectopic expressing vector, PRMT3, or PRMT3 E335Q. **h**. HEK 293T cells transfected with four HA-tag separate plasmids; whole-cell lysis was collected, followed by Co-IP and immunoblots analyses. **i**. HEK 293T cells transfected with HIF1α WT or HIF1α deletion mutant (deletion of 201-400aa), followed by Co-IP or immunoblots analysis. **j**. Testing the sensitivity and specificity of R282 specific asymmetric dimethylarginine antibody. **k-m**. Cells transfected by vector, PRMT3 or PRMT3 E335Q, followed by Co-IP or immunoblots analysis. **n**. Cells treated by SGC707, followed by Co-IP or immunoblots analysis. All immunoblots were conducted three times, providing same results.

Figure S9. PRTM3-mediated tumorigenesis depended on HIF1α R282 methylation. **a**. HIF1α knocked out by lentivirus, followed by immunoblots analysis. **b**. Lovo cells stably expressing HIF1α WT or HIF1α R282K, MG132 to inhibit HIF1α degradation, followed by immunoblots analysis. **c**. ELISA analysis to assess the VEGFA expression level of conditional mediums in normoxia; **p<0.05. **d, e**. Collecting conditional medium from identified cells, culturing HUVECs with conditional mediums, showing representative images of tube formation or transwell assays. **f**. Collecting conditional medium from identified cells, culturing HUVECs with conditional mediums, CCK8 assay to investigate HUVECs proliferation ability; **p<0.05. **g**. Culturing HUVECs with conditional mediums, followed by PI analysis. **k**. Representative images of IHC assays to assess the microvessel density. Student t-test was used for statistical analysis. All immunoblots were conducted three times, and identical results were found.

Figure S10. Inhibiting of HIF1αR282 methylation depressed tumor progress. **a**. Representative images of IHC assays to assess the micro-vessel density. **b**. Amino acid sequence of MPG-peptides. **c, d**. Cells were treated with MPG-peptides, Co-IP and immunoblots analyses to investigate the expression of identified proteins. **e**. Cells treated by MPG-peptides, ELISA assays to assess VEGFA expression level of mediums; **p<0.05. **f**. Collecting conditional mediums from cells respectively treated with MPG-peptides, culturing HUVECs with conditional mediums, followed by CCK8 analysis; **p<0.05. **g**. Representative images of IHC assays to assess the microvessel density. Student t-test was used for statistical analysis. All immunoblots were conducted three times, and identical results were found.
