# Supplementary figures and images for: PRMT3 promotes tumorigenesis by methylating and stabilizing HIF1α in colorectal cancer
Source: Cell Death Dis. 2021 Nov 9;12(11):1066. doi: 10.1038/s41419-021-04352-w (PMC8578369; doi:10.1038/s41419-021-04352-w)

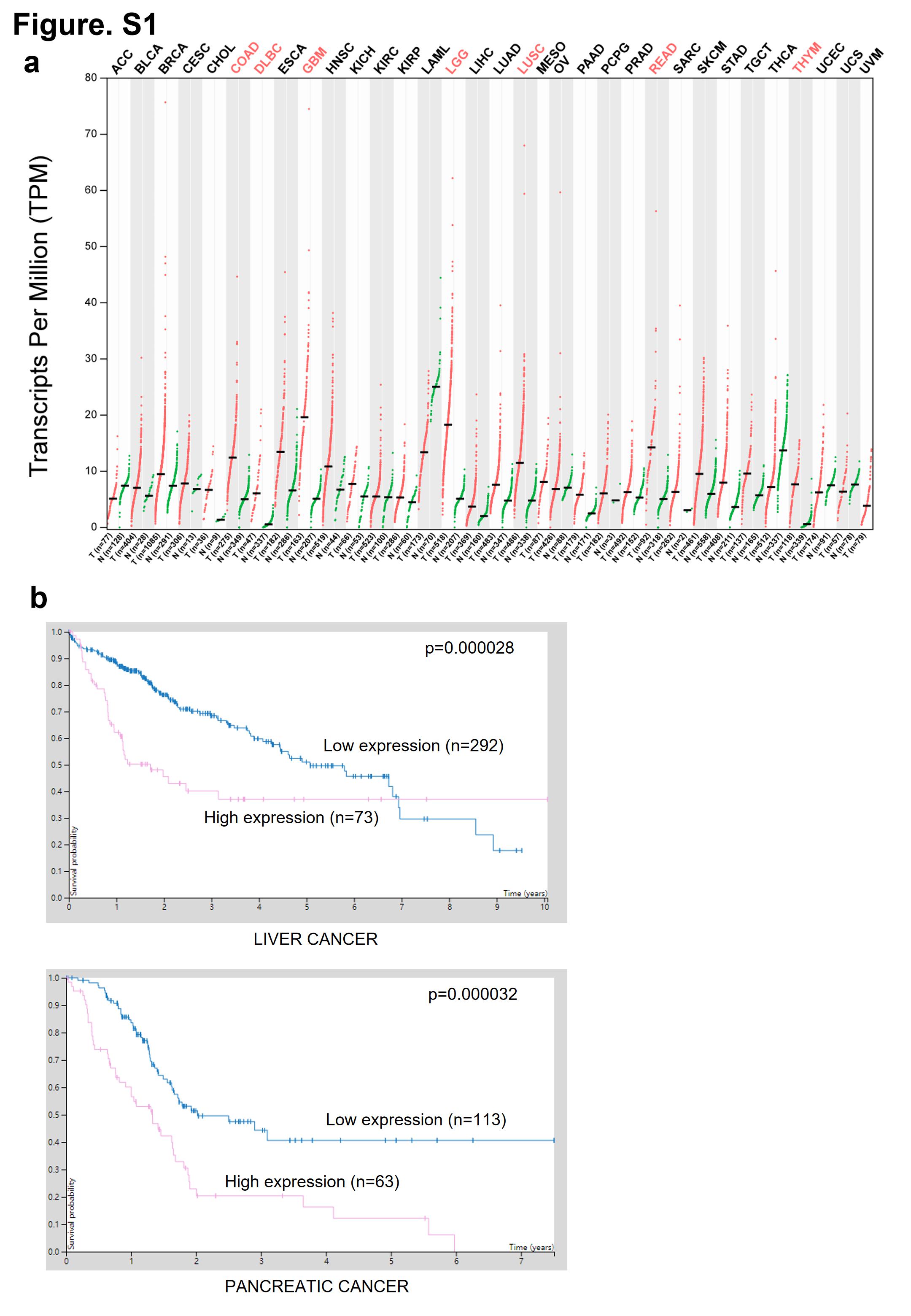

Supplement: Supplementary file 2 — supplementary figure 1 [file 41419_2021_4352_MOESM2_ESM.jpg]

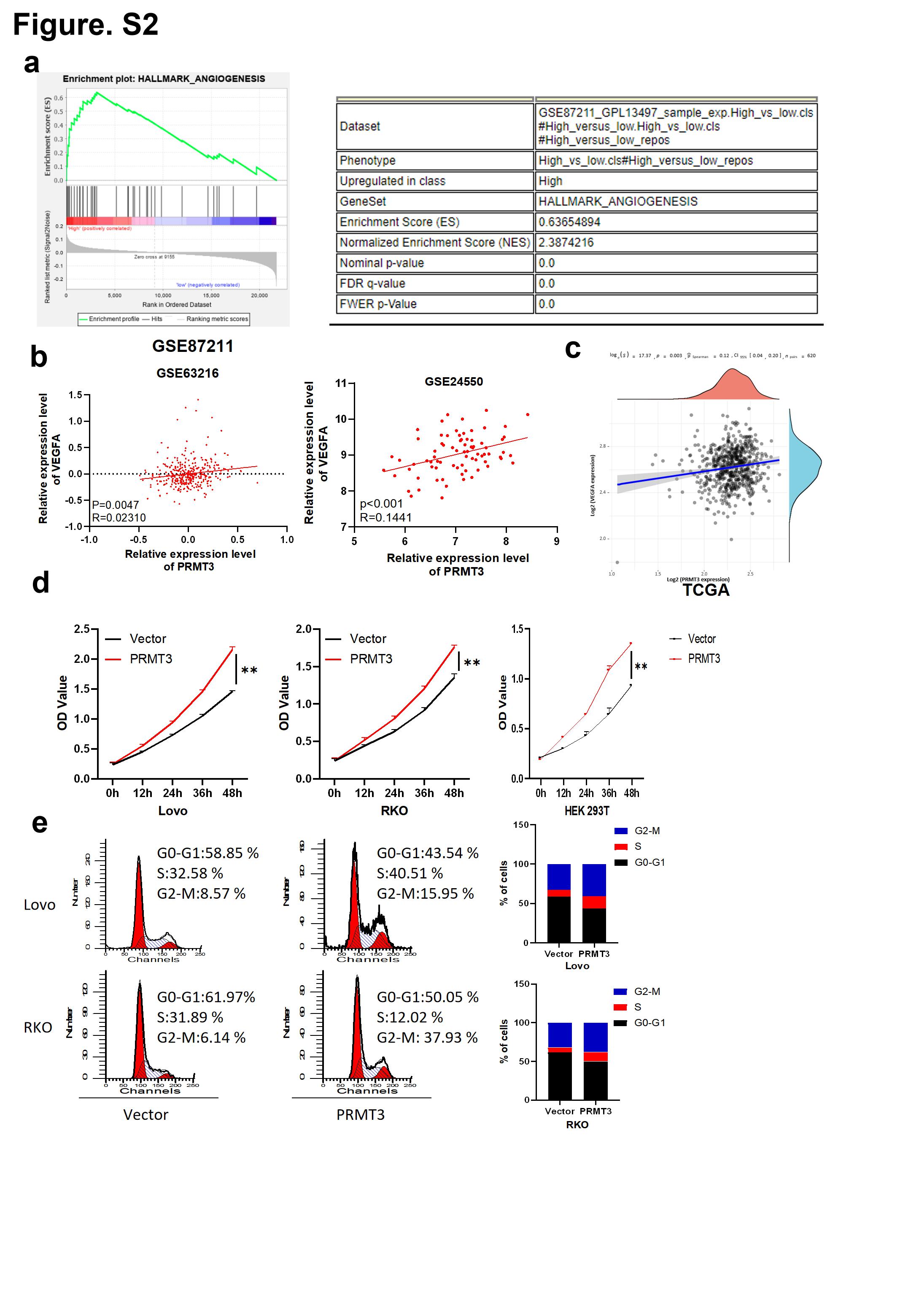

Supplement: Supplementary file 3 — supplementary figure 2 [file 41419_2021_4352_MOESM3_ESM.jpg]

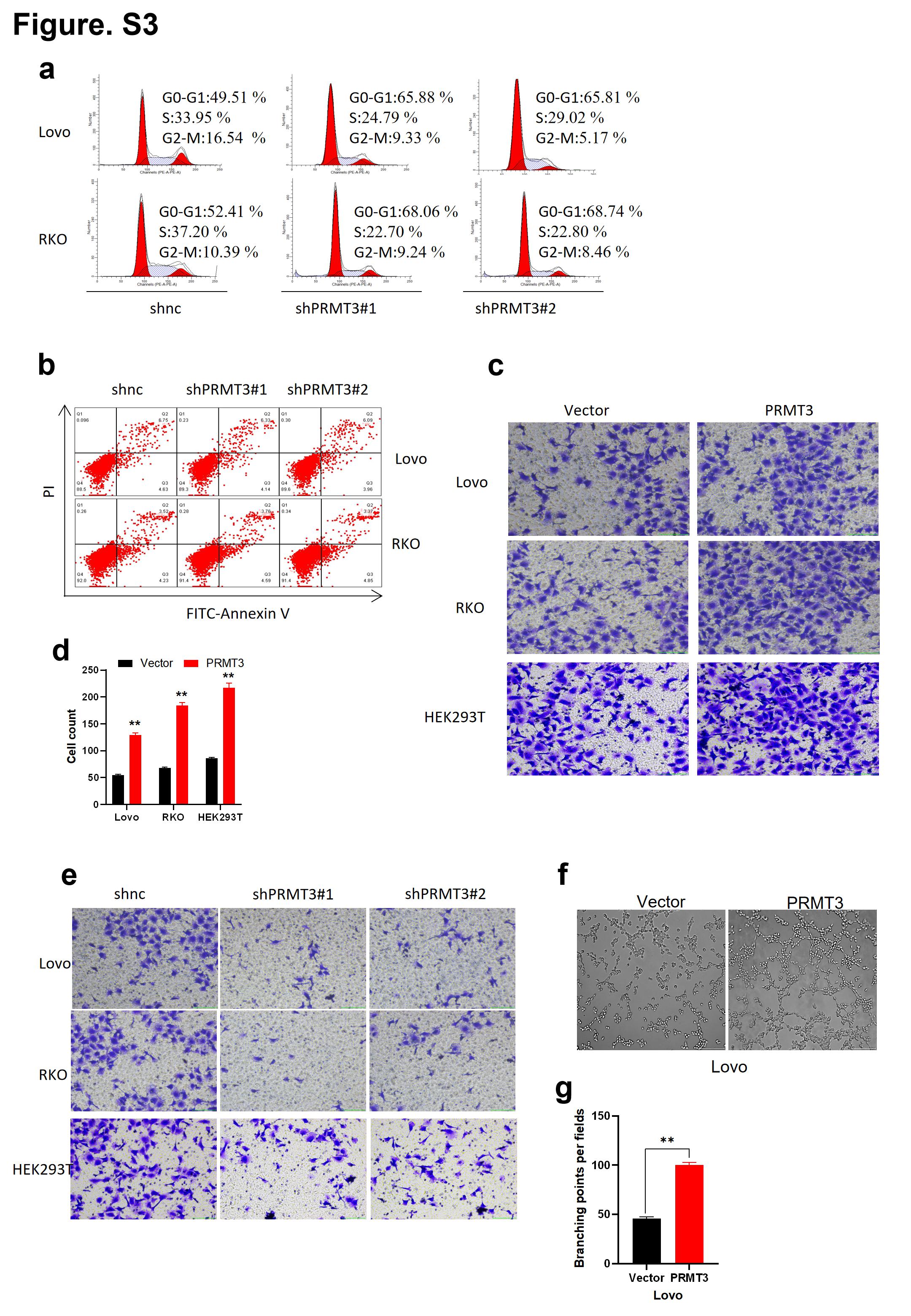

Supplement: Supplementary file 4 — supplementary figure 3 [file 41419_2021_4352_MOESM4_ESM.jpg]

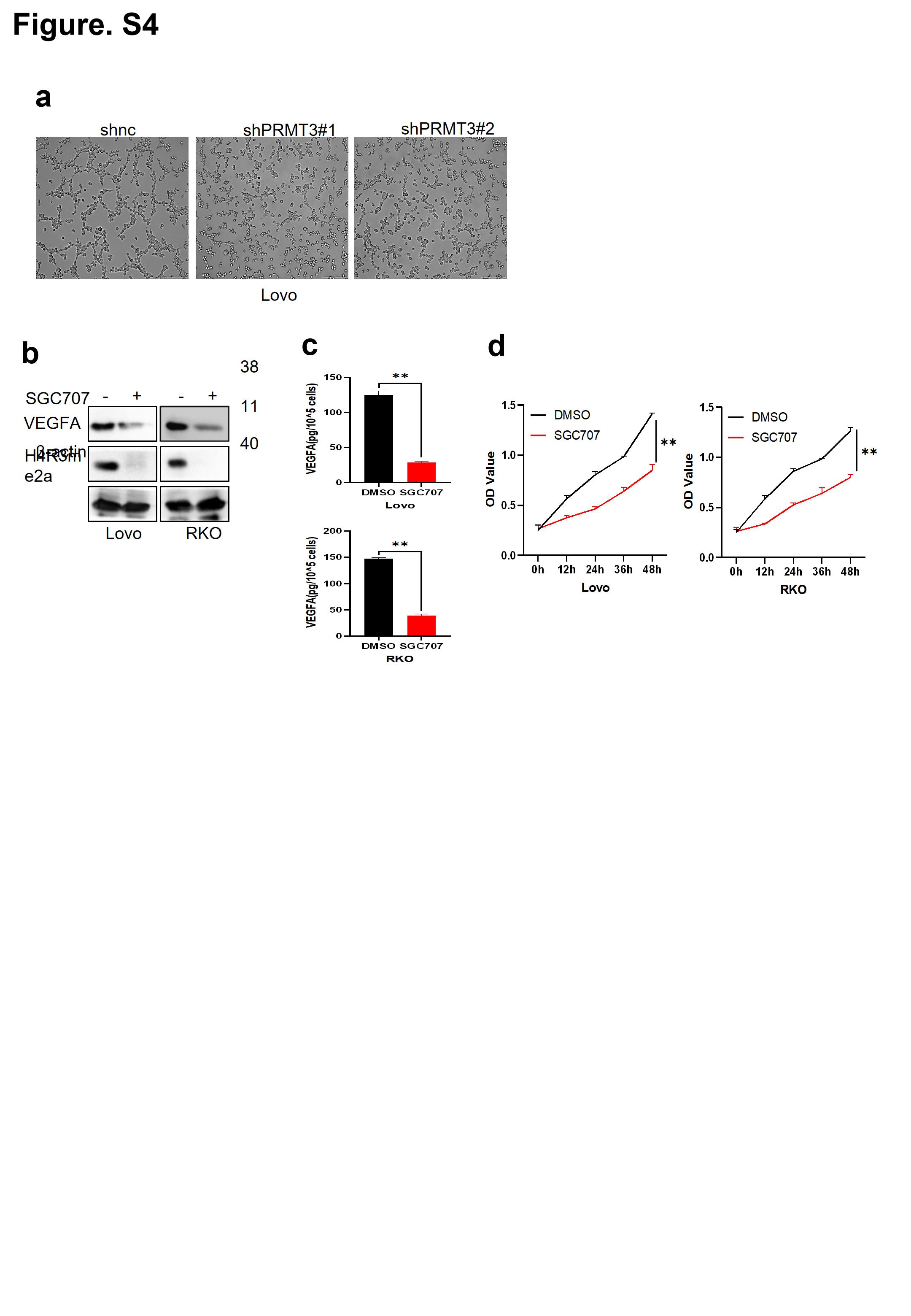

Supplement: Supplementary file 5 — supplementary figure 4 [file 41419_2021_4352_MOESM5_ESM.jpg]

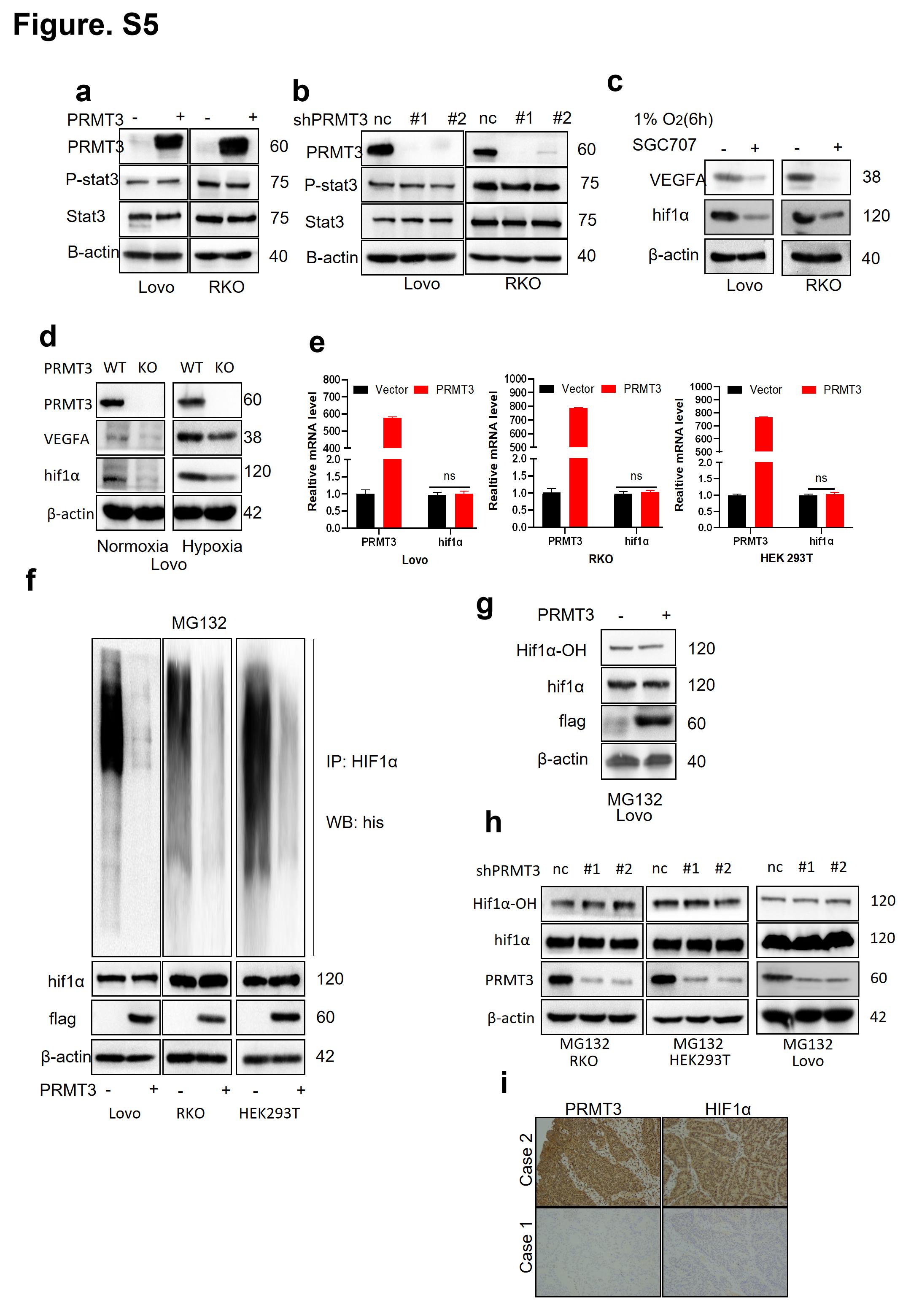

Supplement: Supplementary file 6 — supplementary figure 5 [file 41419_2021_4352_MOESM6_ESM.jpg]

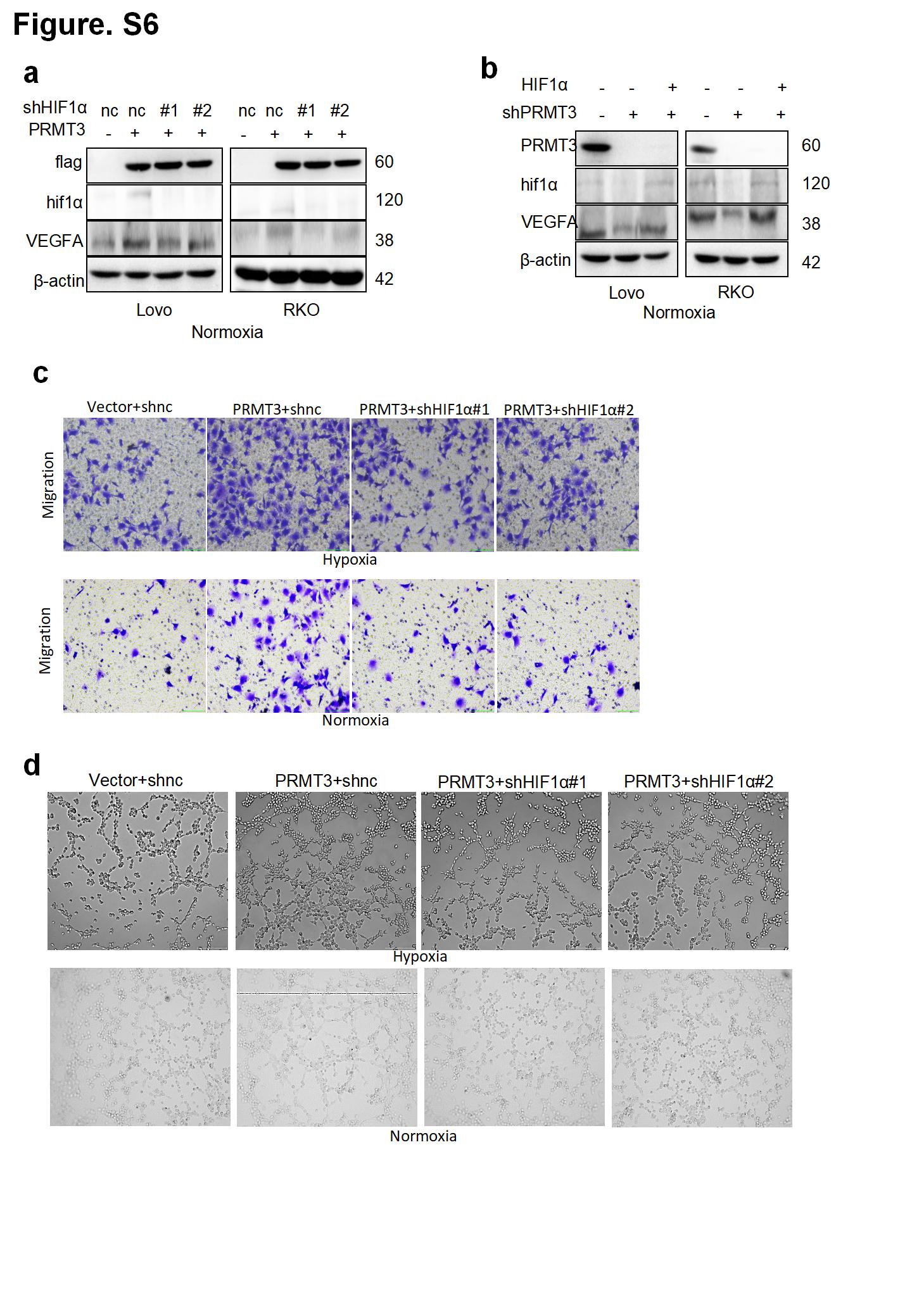

Supplement: Supplementary file 7 — supplementary figure 6 [file 41419_2021_4352_MOESM7_ESM.jpg]

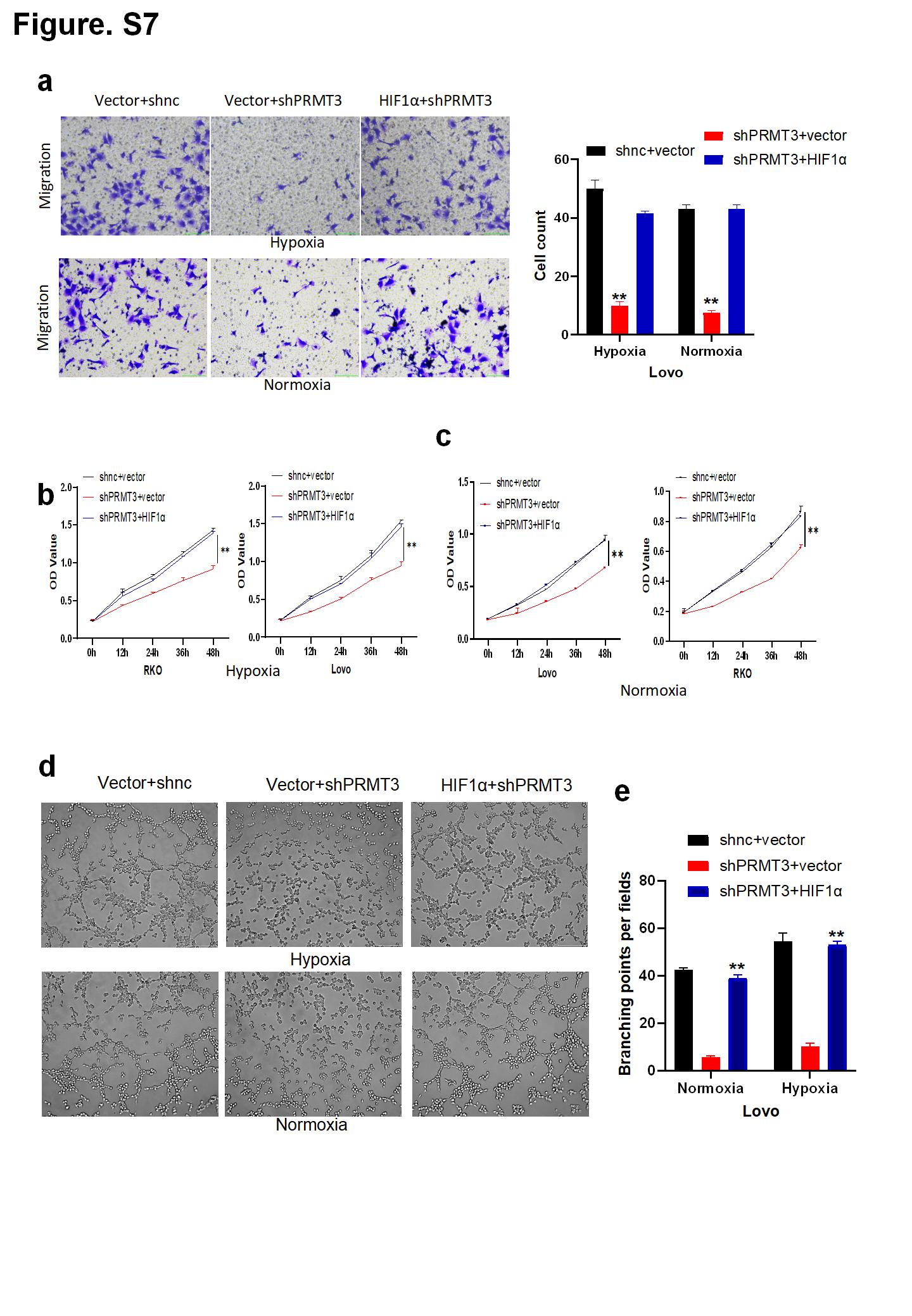

Supplement: Supplementary file 8 — supplementary figure 7 [file 41419_2021_4352_MOESM8_ESM.jpg]

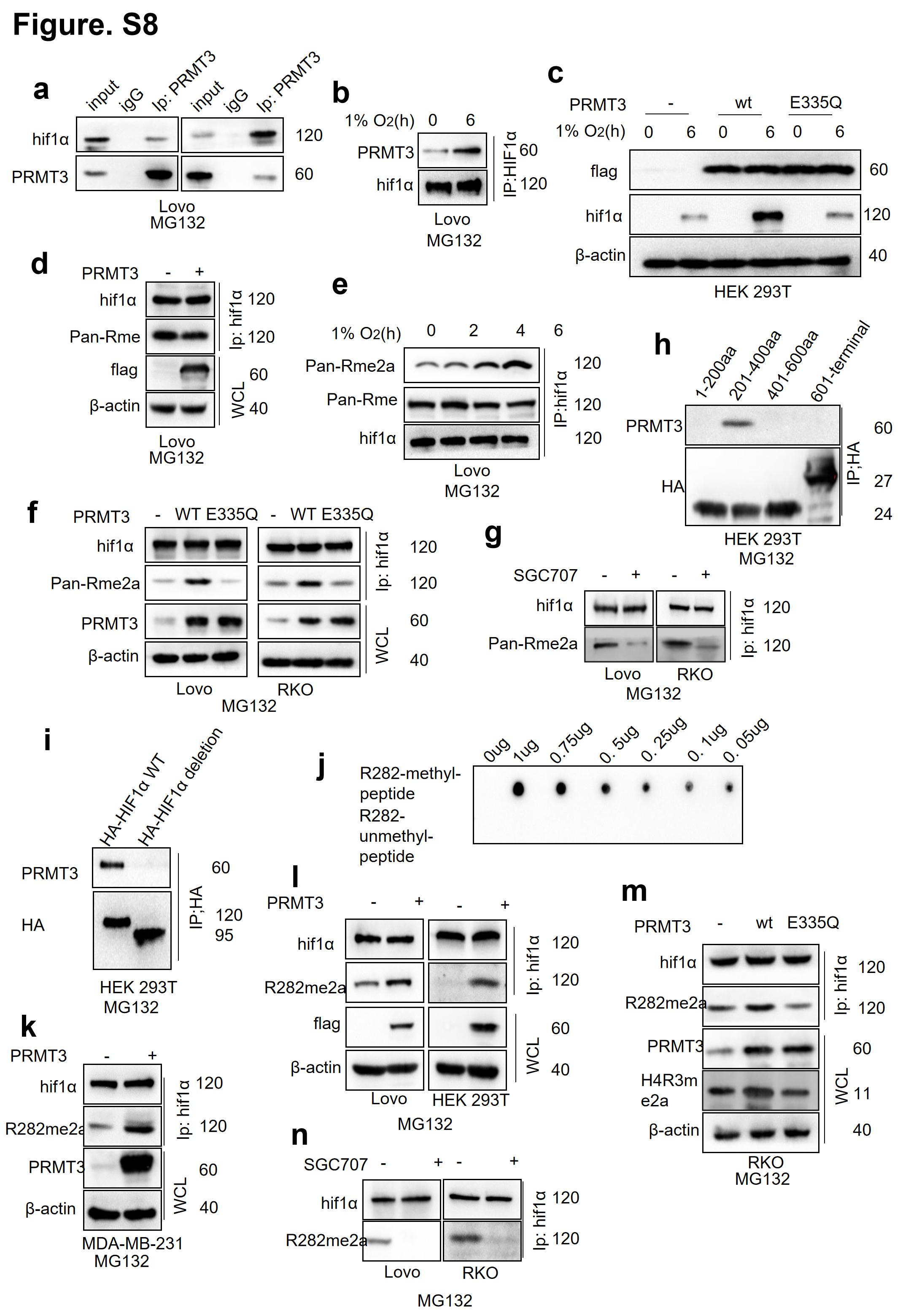

Supplement: Supplementary file 9 — supplementary figure 8 [file 41419_2021_4352_MOESM9_ESM.jpg]

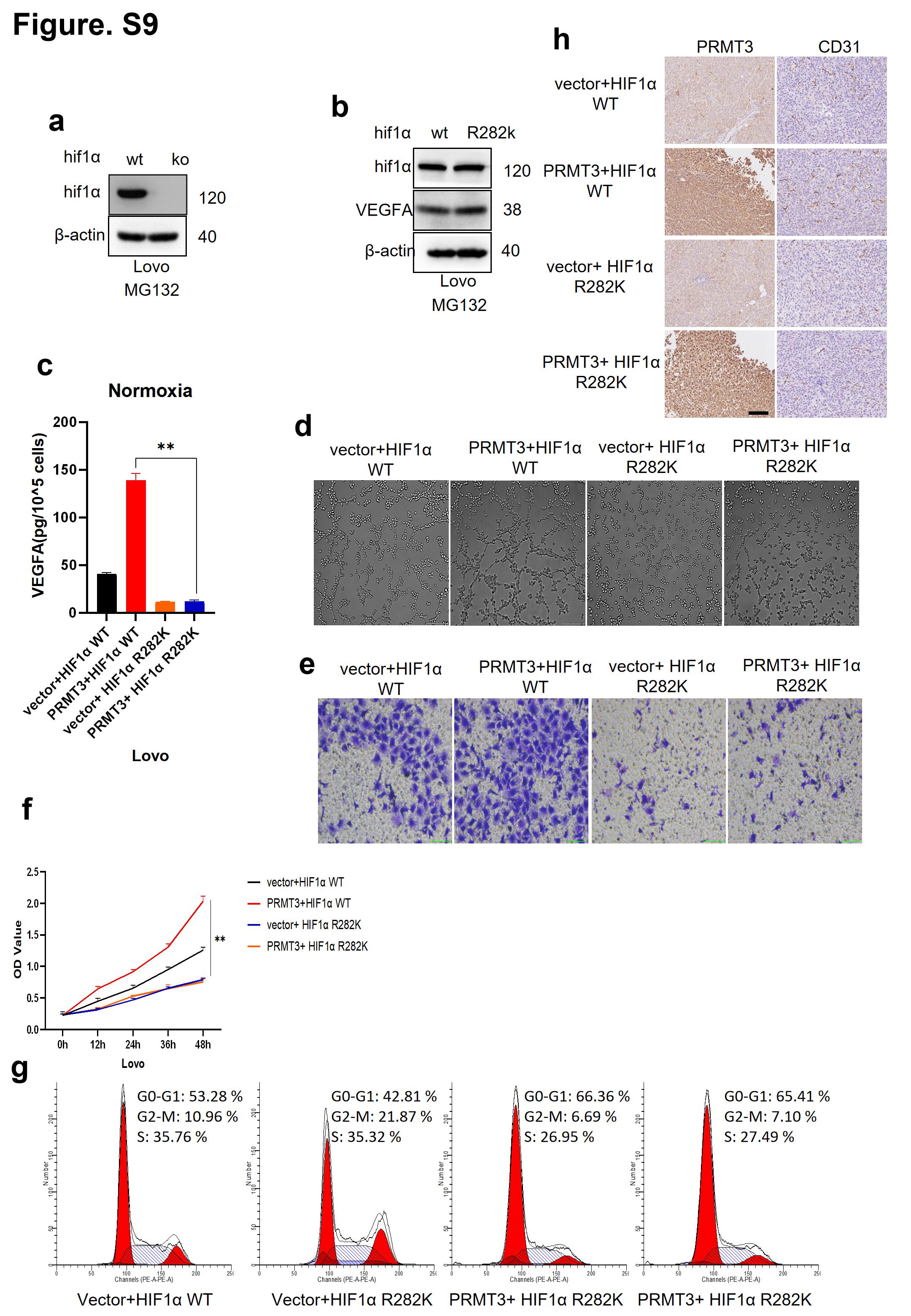

Supplement: Supplementary file 10 — supplementary figure 9 [file 41419_2021_4352_MOESM10_ESM.jpg]

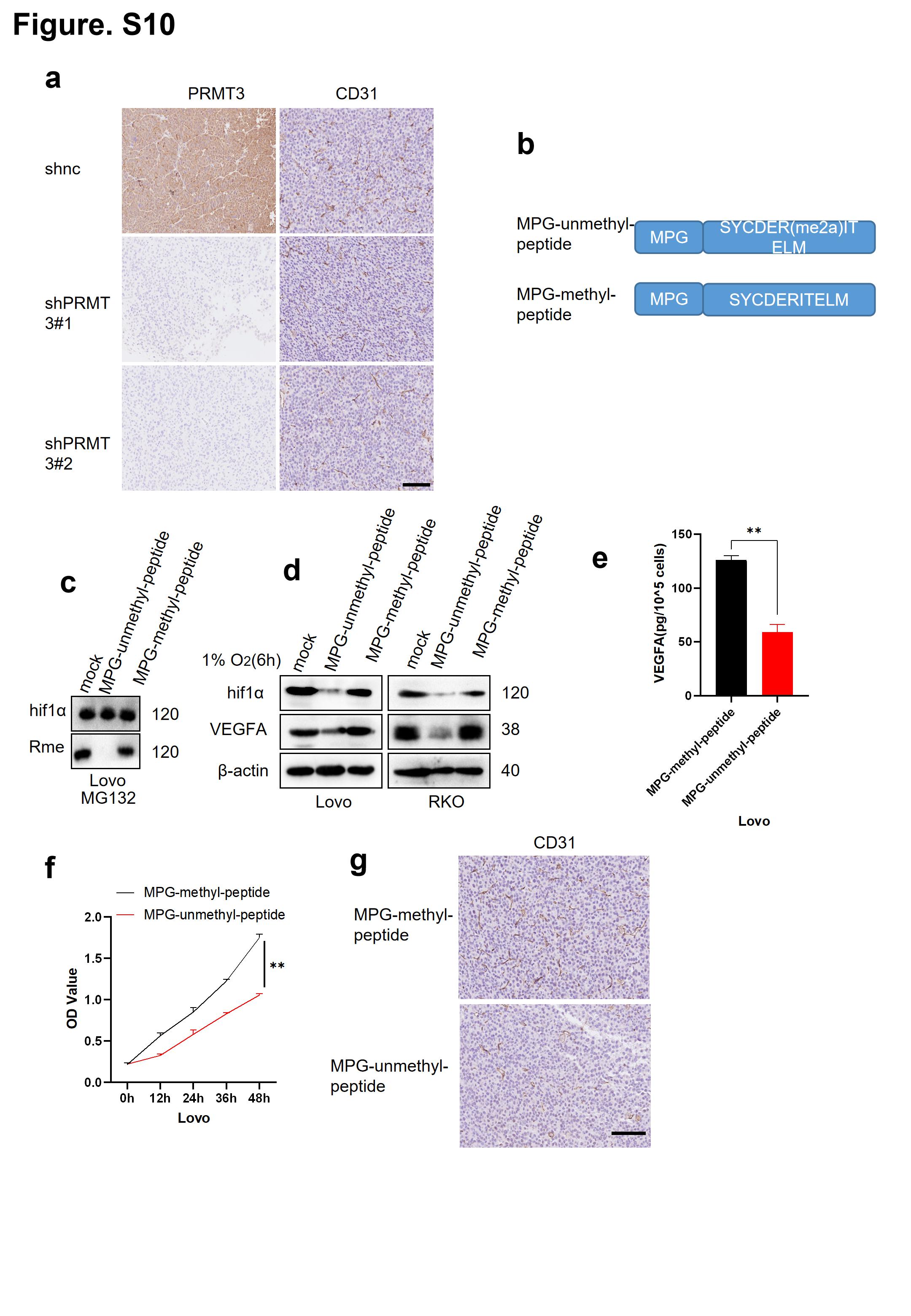

Supplement: Supplementary file 11 — supplementary figure 10 [file 41419_2021_4352_MOESM11_ESM.jpg]
