# Supplementary material for: PRMT3 promotes tumorigenesis by methylating and stabilizing HIF1α in colorectal cancer
Source: Cell Death Dis. 2021 Nov 9;12(11):1066. doi: 10.1038/s41419-021-04352-w (PMC8578369; doi:10.1038/s41419-021-04352-w)
Supplement: Supplementary file 12 — supplementary table 1 [file 41419_2021_4352_MOESM12_ESM.docx]

| Supplementary Table1.The relative expression of indicated genes. | | | | | | | | |
| --- | --- | --- | --- | --- | --- | --- | --- | --- |
| **GENE_ID** | **Description** | **shnc1** | **shnc2** | **shnc3** | **shPRMT3#1** | **shPRMT3#2** | **shPRMT3#3** | **P_value** |
| CCND2 | G1/S-specific cyclin-D2 | 6.38 | 6.14 | 5.96 | 6.70 | 6.67 | 7.05 | 0.020847 |
| CXCL6 | C-X-C motif chemokine 6 | 5.62 | 7.44 | 6.29 | 2.51 | 2.77 | 2.57 | 0.002021 |
| FGFR1 | Fibroblast growth factor receptor 1 | 5.69 | 5.81 | 5.78 | 3.78 | 4.11 | 4.04 | 7.65E-05 |
| ITGAV | Integrin alpha-V | 7.70 | 6.24 | 7.45 | 3.84 | 3.09 | 3.02 | 0.001889 |
| JAG1 | Protein jagged-1 | 5.69 | 6.49 | 6.61 | 8.12 | 7.11 | 8.44 | 0.030056 |
| KCNJ8 | ATP-sensitive inward rectifier potassium channel 8 | 7.57 | 7.16 | 5.44 | 4.54 | 4.75 | 4.21 | 0.029234 |
| LPL | Lipoprotein lipase | 6.70 | 7.59 | 7.06 | 5.41 | 4.53 | 4.75 | 0.003892 |
| LRPAP1 | Alpha-2-macroglobulin receptor-associated protein | 7.91 | 8.30 | 8.05 | 8.68 | 9.04 | 9.43 | 0.016614 |
| LUM | Lumican | 3.45 | 3.16 | 3.34 | 1.44 | 1.65 | 1.70 | 0.000123 |
| MSX1 | Homeobox protein MSX-1 | 8.76 | 8.83 | 7.00 | 3.17 | 3.58 | 3.88 | 0.001824 |
| PDGFA | Platelet-derived growth factor subunit A | 6.23 | 5.83 | 6.42 | 4.81 | 4.60 | 4.35 | 0.002 |
| PGLYRP1 | Peptidoglycan recognition protein 1 | 5.10 | 5.05 | 5.24 | 3.57 | 3.56 | 3.55 | 9.69E-06 |
| POSTN | Periostin | 4.02 | 3.83 | 3.17 | 1.73 | 1.33 | 1.76 | 0.002108 |
| PTK2 | Focal adhesion kinase 1 | 5.25 | 5.50 | 5.36 | 2.14 | 2.90 | 2.99 | 0.000661 |
| STC1 | Stanniocalcin-1 | 6.24 | 6.10 | 5.90 | 2.46 | 2.95 | 2.38 | 6.88E-05 |
| TIMP1 | Metalloproteinase inhibitor 1 | 3.87 | 3.55 | 4.12 | 2.72 | 2.51 | 2.86 | 0.004072 |
| VCAN | Versican core protein | 5.87 | 6.25 | 6.23 | 5.38 | 5.52 | 5.21 | 0.008411 |
| VEGFA | Vascular endothelial growth factor A | 5.97 | 6.21 | 5.96 | 3.81 | 3.71 | 2.33 | 0.004731 |
